# Attempted Suicide Is Independently Associated with Increased In-Hospital Mortality and Hospital Length of Stay among Injured Patients at Community Tertiary Hospital in Japan: A Retrospective Study with Propensity Score Matching Analysis

**DOI:** 10.3390/ijerph21020121

**Published:** 2024-01-23

**Authors:** Yuko Ono, Tokiya Ishida, Nozomi Tomita, Kazushi Takayama, Takeyasu Kakamu, Joji Kotani, Kazuaki Shinohara

**Affiliations:** 1 Department of Disaster and Emergency Medicine, Graduate School of Medicine, Kobe University, Kobe City 650-0017, Japan; takayamaka.zushi@gmail.com (K.T.); kotanijo@med.kobe-u.ac.jp (J.K.); 2 Department of Anesthesiology, Ohta General Hospital Foundation, Ohta Nishinouchi Hospital, Koriyama City 963-8558, Japan; toki007-lightning@poem.ocn.ne.jp (T.I.); tnozomi1129@gmail.com (N.T.); k-shinohara@ohta-hp.or.jp (K.S.); 3 Department of Hygiene and Preventive Medicine, School of Medicine, Fukushima Medical University, Fukushima City 960-1295, Japan; bamboo@fmu.ac.jp

**Keywords:** healthcare resources, prehospital length of stay, severe trauma, suicide attempt

## Abstract

Suicide is an increasingly important public healthcare concern worldwide. Studies examining the effect of attempted suicide on clinical outcomes among patients with trauma are scarce. We conducted a retrospective cohort study at a community emergency department in Japan. We included all severely injured patients with an Injury Severity Score > 15 from January 2002 to December 2021. The primary outcome measure was in-hospital mortality. The other outcome of interest was hospital length of stay. One-to-one propensity score matching was performed to compare these outcomes between suicide attempt and no suicide attempt groups. Of the 2714 eligible patients, 183 (6.7%) had trauma caused by a suicide attempt. In the propensity score-matched analysis with 139 pairs, the suicide attempt group showed a significant increase in-hospital mortality (20.9% vs. 37.4%; odds ratio 2.27; 95% confidence intervals 1.33–3.87) compared with the no suicide attempt group. Among survivors, the median hospital length of stay was significantly longer in the suicide attempt group than that in the no suicide attempt group (9 days vs. 12 days, *p* = 0.0076). Because of the unfavorable consequences and potential need for additional healthcare, increased attention should be paid to patients with trauma caused by a suicide attempt.

## 1. Introduction

Suicide is a major public health issue worldwide. According to a report by the World Health Organization, more than 700,000 people die by suicide every year globally, accounting for more than one in every 100 deaths (1.3%) in 2019 [1]. In Japan, rates of suicide have continued to increase since the coronavirus disease pandemic [2], with >20,000 people dying by suicide in 2022 [3]. Japan had the fourth-highest suicide rate among Organization for Economic Co-operation and Development countries in 2021 [4]. Thus, suicide is a growing global concern and is a particularly urgent issue in Japan.

Among various modalities of suicide attempts, self-inflicted injury is common, and involves a substantial economic burden for society. For example, among Japanese junior and senior high-school students, approximately 10% of respondents reported at least one experience of self-injury [5]. A recent study in the United States (US) reported that the estimated national cost of self-injury mortality rose from $0.46 trillion to $1.12 trillion over the past two decades [6].

Despite the significant societal impact of attempted suicide, studies examining the effect of suicide attempts on clinical outcomes among the trauma population are scarce [7,8,9,10]. Several studies have suggested that patients with trauma caused by a suicide attempt exhibited increased in-hospital mortality compared with those who did not, even after adjusting for potential confounders such as age, sex, vital signs, and injury severity [7,8]. However, several other studies reported no differences between the two groups [9,10]. Given these conflicting findings, the association between a suicide attempt and clinical consequences among injured patients remains unclear, and clarifying this issue will require future studies examining various settings. In addition, no previous studies examining this topic have captured relevant information, such as anatomical region; presentation time (daytime or nighttime); presentation day (weekday or weekend); need for emergency surgery; comorbidities; or diagnosed mental illness [7,8,9,10]. These variables may be important confounders.

Therefore, using our trauma database, which prospectively captures such variables, and propensity score (PS) matching analysis, which is an established method for reducing the effects of confounding factors in a retrospective study, we sought to clarify the association between a suicide attempt and clinical consequences, such as in-hospital mortality and hospital length of stay (LOS) among injured patients at a community tertiary medical center in Japan. We postulated that a suicide attempt would be independently associated with higher rates of in-hospital mortality and longer hospital stays among patients with trauma.

## 2. Materials and Methods

### 2.1. Study Design and Setting

This was a retrospective cohort study at a community emergency and critical care medical center in Japan. Annually, the emergency department (ED) receives > 5000 ambulances and >1200 trauma patients. Of these, approximately 25% are classified as having severe injury, with an Injury Severity Score (ISS) > 15. The facility is the only tertiary and referral medical center within a 50 km radius containing approximately 500,000 residents. At most tertiary hospitals in Japan, including our own, patients who have trauma caused by suicide attempts and are brought to the ED, are initially evaluated by an emergency medical team consisting of attending emergency physicians, emergency medicine residents, post-graduate year 1 or 2 junior residents, and nurses. After the initial resuscitations, a psychiatric consultation is provided by in-house psychiatrists. If patients who have attempted suicide are physically stabilized and need long-term psychiatric care, they are likely to be transferred to psychiatric hospitals.

### 2.2. Participants and Data Sources

After approval by the Institutional Review Board at Ohta Nishinouchi Hospital (approval no. 14_2023), all severely injured patients with ISS > 15 who were transported directly from the scene to the ED between 1 January 2002 and 31 December 2021 were recruited in this study. The board waived the need for patient consent. Patients who received ongoing cardiopulmonary resuscitation at initial contact, patients who were transported from other facilities, and pediatric patients < 15 years of age were excluded from the analysis. Trauma etiology was dichotomized into blunt (e.g., traffic accident, fall, hanging) and penetrating (e.g., stabbing, cutting) injuries. Burn injuries and patients without trauma (such as those affected by self-poisoning) were not included in our trauma database.

Data were collected from a hospital-based electronic database, which prospectively captures each patient’s age; sex; comorbidities; diagnosed mental illness; initial recorded vital signs; physician-staffed ambulance dispatch; need for emergency endotracheal intubation and emergency surgery; ED presentation date and time; prehospital LOS (time from the emergency call to ED arrival); and hospital LOS (time from hospital admission to hospital discharge or transfer). A board-certified emergency physician who specialized in trauma care (author K.S.) scored the Abbreviated Injury Scale (AIS) of each body region, the ISS [11], the Revised Trauma Score (RTS) [12], and the probability of survival using the Trauma and Injury Severity Scores method [13]. To reduce the risk of biased assessment, the author who scored these trauma parameters did not participate in any of the statistical analyses.

### 2.3. Exposures and Outcome Measurement

The patients were classified into suicide attempt and no suicide attempt groups. The no suicide attempt group consisted of patients who had accidental trauma, whereas the suicide attempt group consisted of those who had trauma caused by patients themselves as a result of suicidal intent. Suicide attempts were determined via self-report by the patient, police report, or circumstantial evidence, such as the presence of a suicide note. The primary outcome measure was in-hospital mortality. The other outcomes of interest were prehospital LOS, and hospital LOS among survivors. This study adopted prehospital LOS as a trauma care parameter because prolonged prehospital LOS was known to be associated with poor outcomes of injured patients [14,15,16,17]. Many previous studies similarly considered prehospital LOS to be an important parameter of trauma care [14,15,16,17]. Hospital LOS was also deemed to be a relevant care parameter reflecting increased healthcare resources and costs [7,8,9,10,18,19,20].

Differences in discharge disposition (psychiatric hospital transfer and long-term care facility transfer) among survivors were also compared between patients with self-inflicted injuries and those with unintentional injuries.

### 2.4. Statistical Analysis

A statistical analysis plan was determined a priori. Both crude and matching analyses were performed between the suicide attempt and no suicide attempt groups. Matching analysis was conducted using greedy nearest neighbor one-to-one PS matching without replacement. Multivariable logistic regression was used to find PS to predict the probability of being assigned to the self-inflicted group. In addition to age and sex, the Charlson Comorbidity Index [21,22]; diagnosed mental illness; presentation time and period (8:00–16:59, 17:00–23:59, and 24:00–7:59, weekend or weekday and 2002–2006, 2007–2011, 2012–2016, and 2017–2021, respectively); season (spring: March–May, summer: June–August, autumn: September–November, and winter: December–February); initial recorded vital signs (Glasgow Coma Scale [GCS] score; systolic blood pressure [SBP] and respiratory rate); trauma etiology (blunt or penetrating); injury distribution with AIS ≥ 3; and ISS were selected as explanatory variables for the logistic regression. A set of these variables was chosen a priori on the basis of previous information and biological plausibility. The ISS and AIS are widely used anatomical scoring systems that are correlated with trauma mortality and morbidity [11,12,13]. A high Charlson Comorbidity Index was also known to be associated with increased mortality in patients with trauma [23,24]. Therefore, these variables were incorporated into the logistic regression model to find the PS. To maximize model fitting, patients’ physiological parameters including GCS score, SBP, and respiratory rate were categorized (GCS score: 3, 4–5, 6–8, 9–12, and > 12; SBP: 1–49, 50–75, 76–89, and > 89 mmHg; and respiratory rate: > 29, 10–29, 6–9, 1–5, and 0 breaths/min) according to the scoring system of the RTS [12]. The Charlson Comorbidity Index was also divided into four groups (0, 1, 2, and ≥3). Because our study period was relatively long, the data were divided into four phases (2002–2006, 2007–2011, 2012–2016, and 2017–2021) and each phase was considered as a possible confounder. A previous report employed a similar adjustment strategy [25,26]. Mental illness, season, nighttime, and weekend admission were also incorporated into our model, as described above, because these variables are known to be associated with both suicide attempts and trauma outcomes [27,28,29,30]. All categorical variables mentioned above were dummy coded and incorporated into the PS model. The Hosmer–Lemeshow test and the c-statistic were used to confirm the goodness of fit and discrimination ability of the models. The standardized difference (SD) was used to evaluate the covariate balance; an absolute SD of >10% represents meaningful imbalance [31].

Each patient in the suicide attempt group was matched with a patient in the no suicide attempt group, with the nearest estimated propensity on the logit scale within a specified range (0.2 of the pooled standard deviation of estimated logits) to reduce characteristic differences between the two groups. If two or more patients in the no suicide attempt group met this criterion, one patient was randomly selected for matching. The Mann–Whitney U test was used to compare the prehospital and hospital LOS between the two groups. Chi-squared tests were used to compare hospital mortality between the two groups. All statistical analyses were performed using SPSS Statistics for Windows, version 25.0 (IBM Corp., Armonk, NY, USA). A *p*-value of <0.05 was considered to indicate statistical significance. The violin plots were generated using GraphPad Prism 9 (GraphPad Software, San Diego, CA, USA).

### 2.5. Subanalysis

To evaluate the robustness of the PS-matching analysis described above, inverse probability of treatment weighting (IPTW) analysis was also conducted for hospital mortality. An unconditional logistic regression model adjusted for PS was also fitted using hospital mortality as a dependent variable.

### 2.6. Power Analysis

The retrospective nature of the study predetermined the sample size. The observed power was computed post hoc using G*Power 3 for Windows (Heinrich Heine University, Dusseldorf, Germany) for all primary outcomes examined.

## 3. Results

### 3.1. Participant Flow

**Figure 1:**
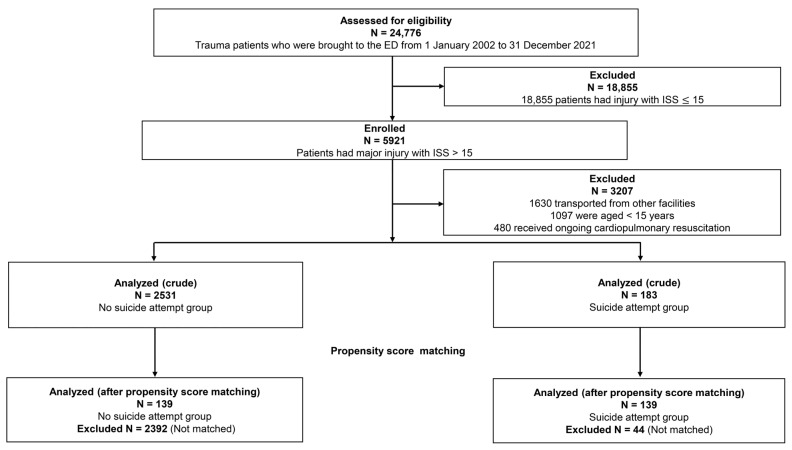
Flow chart showing selection process for injured patients included in analyses. ED, emergency department; ISS, Injury Severity Score.

During the study period, 24,776 trauma patients were transported to the ED, of whom 5921 (23.9%) had severe injury with ISS > 15 (Figure 1). Among them, 1630 patients who were transported from other facilities; 1097 patients < 15 years of age; and 480 patients who received ongoing cardiopulmonary resuscitation were excluded from analysis. The remaining 2714 patients were included in the crude analysis. Of these, 183 (6.7%) had trauma caused by a suicide attempt. Using one-to-one PS-matching, 139 pairs of injured patients who had attempted suicide or not were selected. Complete records were available for all patients, and no data were missing from the analyses. The c-statistic for goodness of fit was 0.92 (95% confidence interval [CI] 0.90–0.94) in the PS model, and the Hosmer–Lemeshow test verified the good fit (*p* = 0.945) of the PS model. Supplementary Figure S1 shows the distributions of PS in the full and matched cohorts.

### 3.2. Characteristics of Study Participants

Table 1 shows the characteristics of all patients (n = 2714) and PS-matched patients (n = 278). The suicide attempt group was more likely to be younger and more likely to be male. There were also statistically significant differences in vital signs such as the GCS score, SBP, and respiratory rate between the two groups. All of these physiological parameters were more severe in the suicide attempt group compared with the no suicide attempt group. Similarly, patients in the suicide attempt group were more likely to have penetrating injuries and severe injuries, with a higher ISS and a higher proportion with AIS ≥ 3 of the head or neck, chest, and extremities or pelvic girdle compared with patients in the no suicide attempt group. Compared with the no suicide attempt group, the suicide attempt group were more likely to present between 24:00–7:59 (35.5% vs. 18.3%) and more likely to receive emergency endotracheal intubation (58.5% vs. 25.1%). After PS-matching, patient distributions were closely balanced, with all SD < 10% between the two groups.

### 3.3. Primary Outcomes

**Figure 2:**
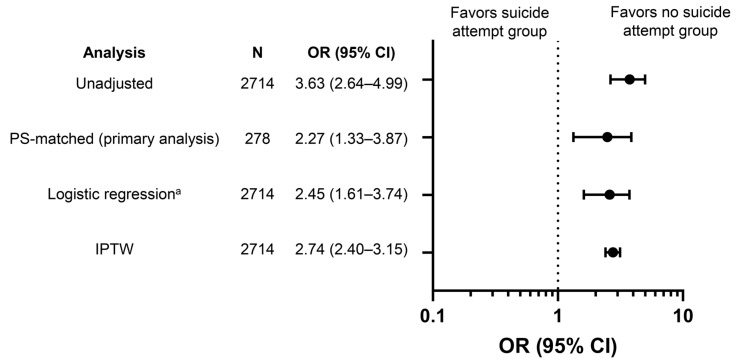
Odds ratios for in-hospital mortality among injured patients: no suicide attempt group vs. suicide attempt group. Reference set was the no suicide attempt group. ^a^ Adjustment for the PS as described in Methods. CI, confidence interval; IPTW, inverse probability of treatment weighting; OR, odds ratio; PS, propensity score.

Figure 2 and Supplementary Table S1 show the differences in-hospital mortality between the no suicide attempt and suicide attempt groups. In PS-matched patients, in-hospital mortality was significantly higher in the suicide attempt group (37.4% vs. 20.9%; odds ratio [OR] 2.27; 95% CI 1.33–3.87). A similar trend was observed with other statistical assumptions, such as in the logistic regression model using PS as an explanatory variable (adjusted OR 2.45; 95% CI 1.61–3.74) and IPTW analysis (OR 2.74; 95% CI 2.40–3.15).

### 3.4. Other Outcomes

**Figure 3:**
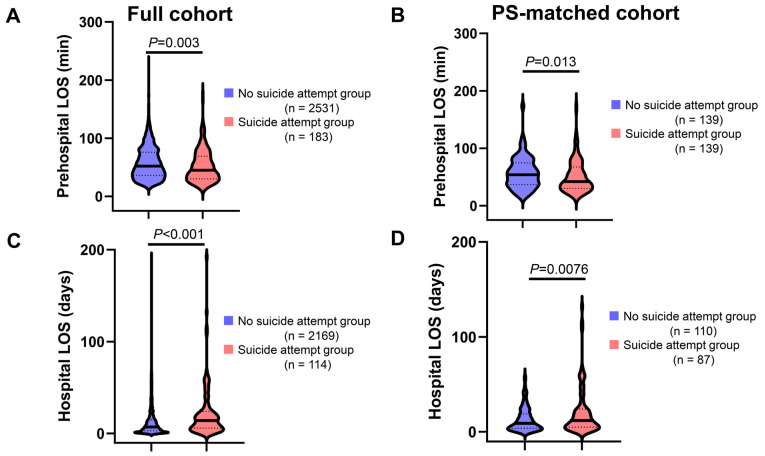
Prehospital and hospital LOS among injured patients: no suicide attempt group vs. suicide attempt group. (**A**,**B**) prehospital LOS in the full (**A**) and PS-matched (**B**) cohort. (**C**,**D**) hospital LOS in the full (**C**) and PS-matched (**D**) cohort. Prehospital LOS is defined as time from the emergency call to ED arrival. Hospital LOS is defined as time from hospital admission to hospital discharge or transfer. Continuous lines and dotted lines within the violin plot indicate median and quartiles, respectively. The *p*-values are derived from the Mann–Whitney U-test. LOS, length of stay; PS, propensity score.

Figure 3A,B show a comparison of prehospital LOS between the suicide attempt and no suicide attempt groups. The median duration of prehospital LOS was significantly shorter in the suicide attempt group than that in the no suicide attempt group in both crude (45.0 min vs. 52.0 min, *p* = 0.003) and PS-matched analyses (42.0 min vs. 54.0 min, *p* = 0.013). Among survivors, the median hospital LOS was significantly longer in the suicide attempt group compared with that in the no suicide attempt group in both the full cohort (14 days vs. 7 days, *p* < 0.001; Figure 3C), and the PS-matched cohort (12 days vs. 9 days, *p* = 0.0076; Figure 3D).

When considering patient disposition among survivors, patients in the suicide attempt group were more likely to be transferred to psychiatric hospitals compared with patients in the no suicide attempt group, both in the full cohort and the PS-matched cohort (Supplementary Table S2). The proportion of patients who transferred to long-term care facilities was similar between the two groups, in both the full cohort and the PS-matched cohort (Supplementary Table S2).

## 4. Discussion

This single-site observational study revealed that a suicide attempt was associated with increased hospital mortality in patients with severe trauma. Among survivors, hospital LOS was also significantly longer in the suicide attempt group compared with the no suicide attempt group. These associations were consistent in both the full cohort and the PS-matched cohort. Our results suggest that patients with severe injury caused by a suicide attempt warrant special attention because of the increased risk of unfavorable consequences and potential need for increased healthcare resources. Thus, the current findings emphasize the importance of prevention of injury caused by suicide attempt.

Our PS-matched analysis indicated that injured patients who attempted suicide had increased hospital mortality compared with those who did not. These associations persisted in other statistical assumptions, such as the IPTW method and a logistic regression model adjusted for PS. Similar associations were also found in two previous studies in mature trauma care systems [7,8]. In Japan, a specialized trauma care system such as that in the US, has not yet been implemented. For example, most Japanese community hospitals, including our study site, do not comply with the American College of Surgeons standards for a level I [32], or even a level II, trauma center [32]. The patient population in the current study differs from those in previous studies in several ways [7,8,9,10]. For example, in previous studies, the proportion of penetrating injury ranged from 28% to 54.1% in the suicide attempt group [7,10]. In contrast, in the current analysis, the rate of penetrating injury in the suicide attempt group was much lower (7%). The current findings corroborate previous reports of an association between increased hospital mortality and suicide attempts, by demonstrating this pattern in a different patient population, geographical region, and healthcare system compared with previous studies [7,8,9,10]. Similar results were observed with different etiologies, such as self-inflicted burn injuries [33,34], supporting the robustness of the association.

There are several plausible explanations for the finding that patients with trauma caused by a suicide attempt had an increased mortality rate compared with those who did not. First, patients with a severe injury caused by a suicide attempt might be subjected to prejudice regarding their medical care [8]. For example, if a patient with trauma caused by a suicide attempt is unconscious, their close relatives may be more likely to choose to withhold or withdraw life-sustaining treatment when discussing resuscitation code status, in consideration of the patients’ intention. Additionally, if a patient who attempted suicide is conscious, they may be less likely to be motivated in their recovery, and they may decline further necessary treatment and rehabilitation. Thus, it is possible that prejudice presents in a variety of ways that ultimately result in a higher mortality for patients with trauma caused by suicide attempt.

Other possible factors contributing to unfavorable outcomes in injured patients who attempted suicide may be related to differences in socioeconomic status. Patients who attempted suicide are more likely to be socially or economically poor; these characteristics are known to be associated with poor survival outcomes among patients with trauma [35,36] and a variety of other conditions [37].

In addition to age, sex, anatomical severity, and physiological severity, the current study captured in-depth information, such as presentation time; presentation day; anatomical site; need for emergency surgery; comorbidities; and diagnosed mental illness. These variables are important potential confounders that were not adjusted for in previous studies [7,8,9,10]. We believe that this strength of our study further supports the independent association between suicide attempts and increased mortality among trauma patients.

In accord with several previous studies [7,9], the current findings indicated that hospital LOS among survivors was significantly longer in the suicide attempt group compared to the no suicide attempt group. It is plausible that social factors, such as difficulty finding a place to be discharged, played a role in this finding. For example, the patient’s family or secondary hospitals without a psychiatric department may be likely to express difficulties over accepting patients who attempted suicide compared with patients who did not. The current findings also revealed that injured patients who attempted suicide were more likely to be transferred to psychiatric hospitals. Physical stabilization is generally a prerequisite for psychiatric hospital transfer in Japan, which takes a relatively long time. It is also plausible that the suicide attempt group was more likely to have psychological symptoms compared with the no suicide attempt group, the presence of which is known to be associated with prolonged hospital stay [38,39]. Previous studies have reported that prolonging hospital LOS occupies beds and caregivers for a longer time, as well as increasing healthcare costs and economic burden [40,41]. The excess length of hospital stay is also known to be associated with increased complications, such as hospital-acquired infections [42,43]. The current findings, taken together with previous reports [7,9], indicate that injured patients who attempted suicide require higher levels of healthcare resources than patients those who did not.

The current findings also revealed that prehospital LOS was shorter in the suicide attempt group compared with the no suicide attempt group. The study facility is the only emergency and critical care medical center within this medical control area, and is expected to receive physically and socially vulnerable cases. Because of this social responsibility, the self-inflicted group may have been admitted by the study facility relatively quickly.

### Limitations and Strengths

Several limitations of the current study should be acknowledged. First, this study was performed at a single site, limiting the generalizability of the findings. Japanese healthcare systems are typically well organized, with minimal variations of sociodemographic conditions across regions [33]. Therefore, it may not be possible to extrapolate our findings to other medical institutions, particularly those in an underdeveloped social environment.

Second, although rigorous adjustments were made in the PS-matched analysis, other unmeasured factors may have confounded our results, as with any observational study. For example, several important covariates, such as distance to the scene, fluid resuscitation, social status, insurance status, and use of vasopressors, were not captured in our database. In addition, although alcohol or psychoactive drug use at the time of the injury can affect the trauma outcome [44,45], our database did not record these variables. Furthermore, although our database captured the time from the emergency call to ED arrival (prehospital LOS), the time from injury occurrence to emergency call was not recorded. Accidental injury is likely to occur in public places, and ambulances are likely to be called immediately by bystanders or patients by themselves. In contrast, a suicide attempt is less likely to occur in a public place, and less likely to be witnessed by bystanders. It is also possible that patients who attempted suicide exhibit hesitation regarding calling ambulances. Such delays may have been present but were not reflected in our outcome measures. Further analyses including these variables will be needed to further clarify the association between injuries caused by suicide attempt and measured clinical outcomes.

Third, it is possible that there were missed or misclassified suicide attempts. We speculate that suicide attempts may have been underestimated because some injured patients may have concealed their attempted suicide. This could have potentially biased our results toward the null hypothesis.

Finally, the sample size was relatively small, and was not determined a priori. As described in the Methods, because of the retrospective nature of the current study, it was not possible to predetermine the sample size. Nevertheless, a post-hoc power calculation indicated that the power in our study was sufficient (power > 0.80) for all primary outcomes examined.

Despite these limitations, the current study had several strengths. First, there were no missing data for all relevant analyses, maximizing the quality of PS-matched analysis. The measured outcomes were objective (i.e., hospital mortality and hospital LOS) and less prone to diagnostic errors. Furthermore, to mitigate the risk of biased assessment, the author who constructed the database (K.S.) was not involved in the any of the statistical analysis. Therefore, we believe that the current study accurately delineated the impact of injury caused by attempted suicide on mortality and use of medical resources in a typical Japanese ED.

## 5. Conclusions

At a community tertiary hospital in Japan, injuries caused by suicide attempts were independently associated with increased mortality compared with other types of injuries. Among survivors, hospital LOS was also significantly longer in trauma patients who attempted suicide compared with those who did not. Increased attention should be paid to this subset of trauma patients regardless of demographic characteristics or severity of injury, because of the more severe consequences and the potential need for additional healthcare resources.

## Figures and Tables

**Table 1:** Demographic and clinical characteristics of injured patients: no suicide attempt group vs. suicide attempt group.

	Full Cohort				PS Matched Cohort			
	No Suicide Attempt (n = 2531)	Suicide Attempt (n = 183)	*p*	SD (%)	No Suicide Attempt (n = 139)	Suicide Attempt (n = 139)	*p*	SD (%)
Age								
Median (interquartile range)	63.0 (46.0–75.0)	40.0 (28.0–58.0)	<0.001	NA	46.0 (31.0–64.0)	46.0 (31.0–62.0)	0.618	NA
Mean ± standard deviation	59.2 ± 20.0	43.2 ± 18.0	<0.001	−84.1	47.2 ± 20.1	46.0 ± 18.2	0.600	−6.3
Sex			<0.001				0.805	
Male	1830 (72.3)	98 (53.6)		−39.5	87 (62.6)	85 (61.2)		−2.9
Female	701 (27.7)	85 (46.4)		39.5	52 (37.4)	54 (38.8)		2.9
Admission phase			0.142				0.974	
2002–2006	529 (20.9)	33 (18.0)		−7.3	21 (15.1)	21 (15.1)		0
2007–2011	598 (23.6) *	57 (31.1) **		16.9	40 (28.8)	43 (30.9)		4.6
2012–2016	734 (29.0)	47 (25.7)		−7.4	41 (29.5)	38 (27.3)		−4.9
2017–2021	670 (26.5)	46 (25.1)		−3.2	37 (26.6)	37 (26.6)		0
Trauma etiology			<0.001				0.642	
Blunt	2515 (99.4)	170 (92.9)		−34.3	130 (93.5)	128 (92.1)		−5.4
Penetrating	16 (0.6)	13 (7.1)		34.3	9 (6.5)	11 (7.9)		5.4
Anatomical severity								
ISS								
Median (interquartile range)	25.0 (18.0–30.0)	30.0 (24.0–45.0)	<0.001	NA	29.0 (20.0–45.0)	29.0 (22.0–41.0)	0.684	NA
Mean ± Standard Deviation	26.7 ± 10.9	35.7 ± 17.1	<0.001	62.8	33.9 ± 17.3	33.3 ± 15.0	0.739	−3.7
AIS (≥3)								
Head or neck	1489 (58.8)	52 (28.4)	<0.001	−64.4	51 (36.7)	45 (32.4)	0.449	−9.1
Face	74 (2.9)	9 (4.9)	0.130	10.3	7 (5.0)	6 (4.3)	0.776	−3.3
Chest	1402 (55.4)	122 (66.7)	0.003	23.3	99 (71.2)	95 (68.3)	0.601	−6.3
Abdomen or pelvic contents	347 (13.7)	30 (16.4)	0.311	7.6	19 (13.7)	19 (13.7)	1.000	0
Extremities or pelvic girdle	560 (22.1)	69 (37.7)	<0.001	34.6	46 (33.1)	49 (35.3)	0.704	4.6
Physiological parameters								
GCS score			<0.001				0.980	
13–15	1811 (71.6) **	104 (56.8) *		−31.2	83 (59.7)	84 (60.4)		1.4
9–12	256 (10.1) *	36 (19.7) **		27.2	24 (17.3)	24 (17.3)		0
6–8	202 (8.0)	13 (7.1)		−3.4	8 (5.8)	10 (7.2)		5.7
4–5	102 (4.0)	9 (4.9)		4.4	7 (5.0)	6 (4.3)		−3.3
3	160 (6.3) *	21 (11.5) **		18.3	17 (12.2)	15 (10.8)		−4.4
SBP, mmHg			<0.001				0.962	
>89	2239 (88.5) **	142 (77.6) *		−29.4	110 (79.1)	113 (81.3)		5.5
76–89	114 (4.5)	12 (6.6)		9.2	11 (7.9)	9 (6.5)		−5.4
50–75	134 (5.3)	14 (7.7)		9.7	11 (7.9)	10 (7.2)		−2.6
1–49	44 (1.7) *	15 (8.2) **		30.3	7 (5.0)	7 (5.0)		0
Respiratory rate, breaths/min			<0.001				0.865	
>29	2110 (83.4) **	123 (67.2) *		−38.2	100 (71.9)	101 (72.7)		1.8
10–29	370 (14.6) *	50 (27.3) **		31.6	30 (21.6)	32 (23.0)		3.4
6–9	30 (1.2)	1 (0.5)		−7.6	2 (1.4)	1 (0.7)		−6.9
1–5	2 (0.08)	0 (0)		−4.0	0 (0)	0 (0)		0
0	19 (0.8) *	9 (4.9) **		24.8	7 (5.0)	5 (3.6)		−6.9
RTS								
Median (interquartile range)	7.841 (6.817–7.841)	7.550 (5.881–7.841)	<0.001	NA	7.550 (5.967–7.841)	7.550 (5.967–7.841)	0.598	NA
Mean ± standard deviation	7.035 ± 1.373	6.451 ± 1.961	<0.001	−34.5	6.524 ± 1.935	6.641 ± 1.834	0.303	6.2
Probability of survival								
Median (interquartile range)	0.932 (0.778–0.962)	0.918 (0.518–0.980)	0.144	NA	0.901 (0.553–0.978)	0.924 (0.695–0.981)	0.408	NA
Mean ± Standard Deviation	0.806 ± 0.265	0.725 ± 0.342	<0.001	−26.5	0.760 ± 0.333	0.766 ± 0.320	0.878	1.8
Charlson Comorbidity Index			<0.001				0.958	
0	2025 (80.0) **	108 (59.0) *		−46.8	92 (66.2)	91 (65.5)		−1.5
1	248 (9.8) *	66 (36.1) **		65.6	39 (28.1)	40 (28.8)		1.6
2	142 (5.6) **	2 (1.1) *		−25.2	3 (2.2)	2 (1.4)		−6.0
≥3	116 (4.6)	7 (3.8)		−4.0	5 (3.6)	6 (4.3)		3.6
Diagnosed mental illness	98 (3.9)	75 (41.0)	<0.001	2.2	43 (30.9)	42 (30.2)	0.896	
Presentation time			<0.001				0.924	
8:00–16:59	1292 (51.0) **	60 (32.8) *		−37.5	49 (35.3)	50 (36.0)		1.5
17:00–23:59	777 (30.7)	58 (31.7)		2.2	45 (32.4)	42 (30.2)		−4.5
24:00–7:59	462 (18.3) *	65 (35.5) **		39.5	45 (32.4)	47 (33.8)		3.2
Presentation day			0.173				0.474	
Weekdays	1818 (71.8)	140 (76.5)		10.8	29 (20.9)	34 (24.5)		8.6
Weekends	713 (28.2)	43 (23.5)		−10.8	110 (79.1)	105 (75.5)		−8.6
Season			0.292				0.999	
Spring (March–May)	625 (24.7)	54 (29.5)		10.8	38 (27.3)	39 (28.1)		1.8
Summer (June–August)	660 (26.1)	52 (28.4)		5.2	37 (26.6)	37 (26.6)		0
Autumn (September–November)	649 (25.6)	40 (21.9)		−8.7	32 (23.0)	31 (22.3)		−1.7
Winter (December–February	597 (23.6)	37 (20.2)		−8.2	32 (23.0)	32 (23.0)		0
Intervention								
Physician-staffed ambulance dispatch	945 (37.3)	79 (43.2)	0.116	12.1	63 (45.3)	61 (43.9)	0.809	−2.8
Emergency endotracheal intubation	635 (25.1)	107 (58.5)	<0.001	72.0	64 (46.0)	70 (50.4)	0.548	8.8
Emergency surgery	412 (16.3)	37 (20.2)	0.166	10.1	30 (21.6)	25 (18.0)	0.452	−9.0

Data are expressed as n (%) unless otherwise noted. ** Adjusted standardized residual > 1.96. * Adjusted standardized residual < −1.96. The *p*-values were derived from the Mann–Whitney U test or chi-squared tests. AIS, Abbreviated Injury Scale; ETI, endotracheal intubation; GCS, Glasgow Coma Scale; ISS, Injury Severity Score; NA, not available; PS, propensity score; RTS, Revised Trauma Score; SBP, systolic blood pressure; SD, standardized difference.

## Data Availability

The minimal anonymous dataset used in this study is included in Supplementary Data S1 in the Supporting Information File.

## Supplementary Materials

The following supporting information can be downloaded at: https://www.mdpi.com/article/10.3390/ijerph21020121/s1, Figure S1. Distribution of propensity score in the full (A) and propensity score-matched (B) cohorts. Table S1. Comparison of mortality rate: no suicide attempt group vs. suicide attempt group. Table S2. Disposition location among survivors: no suicide attempt group vs. suicide attempt group. Data S1. The minimal anonymous dataset used in this study.
