# Supplementary material for: Attempted Suicide Is Independently Associated with Increased In-Hospital Mortality and Hospital Length of Stay among Injured Patients at Community Tertiary Hospital in Japan: A Retrospective Study with Propensity Score Matching Analysis
Source: Int J Environ Res Public Health. 2024 Jan 23;21(2):121. doi: 10.3390/ijerph21020121 (PMC10888049; doi:10.3390/ijerph21020121)
Supplement: Supplementary file 1 [file ijerph-21-00121-s001.zip › ijerph-2750980-supplementary tables and figure.pdf]

Table S1. Comparison of mortality rate: no suicide attempt group vs suicide attempt group.

	Full cohort			PS matched cohort		
	**No suicide attempt (n = 2531)**	**Suicide attempt (n = 183)**	***P***	**No suicide attempt (n = 139)**	**Suicide attempt (n = 139)**	***P***
**Outcome**			< 0.001			0.002
Dead	362 (14.3)	69 (37.7)		29 (20.9)	52 (37.4)	
Alive	2169 (85.7)	114 (62.3)		110 (79.1)	87 (62.6)	

Data are expressed as n (%). PS, propensity score.

Table S2. Disposition location among survivors: no suicide attempt group vs suicide attempt group.

	Full cohort			PS matched cohort		
	**No suicide attempt (n = 2169)[a]**	**Suicide attempt (n = 114)**	**OR (95% CI)**	**No suicide attempt (n = 110) [a]**	**Suicide attempt (n = 87)**	**OR (95% CI)**
Long-term care facility	165 (7.6)	6 (5.3)	0.68 (0.29–1.56)	7 (6.4)	5 (5.7)	0.90 (0.28–2.93)
Psychiatric hospital	3 (0.1)	9 (7.9)	61.9 (16.5–232.0)	1 (0.9)	8 (9.2)	11.0 (1.35–90.0)

[a]The reference set was the no suicide attempt group. Data are expressed as n (%). PS, propensity score.

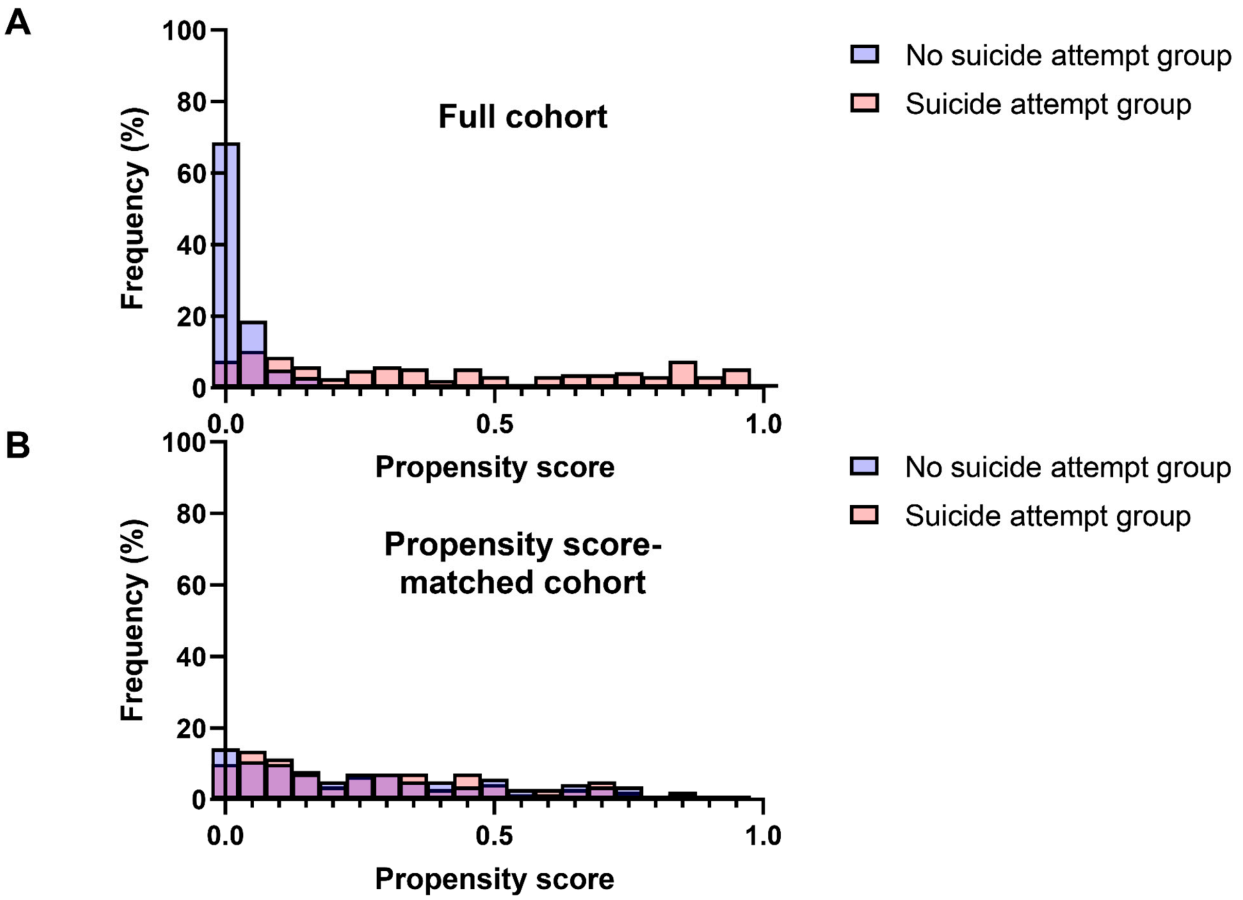

Figure S1. Distribution of propensity score in the full (A) and propensity score-matched (B) cohorts.
